# DARS-AS1 accelerates the proliferation of cervical cancer cells via miR-628-5p/JAG1 axis to activate Notch pathway

**DOI:** 10.1186/s12935-020-01592-2

**Published:** 2020-11-03

**Authors:** Yihong Chen, Qiumei Wu, Jing Lin, Juanbing Wei

**Affiliations:** 1 Department of Obstetrics and Gynecology, The First Affiliated Hospital of Fujian Medical University, No. 20 Chazhong Road, Taijiang District, Fuzhou, 350000 Fujian China; 2 Fujian Provincial Maternal and Child Health Care Hospital, No. 18 Daoshan Road, Gulou District, Fuzhou, 350000 Fujian China; 3 Department of Embryology, Fujian Medical University, No. 88 Jiaotong Road, Taijiang District, Fuzhou, 350000 Fujian China

**Keywords:** DARS-AS1, miR-628-5p, JAG1, Notch signaling pathway, Cervical cancer

## Abstract

**Background:**

Growing evidence has indicated the vital parts of long non-coding RNAs (lncRNAs) in modulating the progression of assorted human cancers, including cervical cancer (CC). Nevertheless, the role and mechanism of aspartyl-tRNA synthetase antisense RNA 1 (DARS-AS1) have been not comprehensively illustrated in CC yet.

**Methods:**

Real-time quantitative polymerase chain reaction (RT-qPCR) was exploited for assessing RNA expression while western blot for protein expression in CC cells. The cell counting kit-8 (CCK-8), colony formation and TdT-mediated dUTP Nick-End Labeling (TUNEL) assays, as well as flow cytometry analysis, were employed to evaluate the modulation of DARS-AS1 on the proliferation and apoptosis of CC cells. In addition, RNA immunoprecipitation (RIP), RNA pull down assay and luciferase reporter assay confirmed the interactivity among DARS-AS1, miR-628-5p and jagged canonical Notch ligand 1 (JAG1). RBP-JK luciferase reporter assay determined the activity of Notch pathway.

**Results:**

DARS-AS1 level was significantly increased in CC cells. Moreover, down-regulation of DARS-AS1 hampered cell the proliferation and accelerated the apoptosis of CC cells. Importantly, DARS-AS1 was a competing endogenous RNA (ceRNA) to elevate JAG1 level through sequestering miR-628-5p, leading to activated Notch pathway to aggravate CC tumorigenesis.

**Conclusions:**

DARS-AS1/miR-628-5p/JAG1/Notch signaling accelerates CC progression, indicating DARS-AS1 as a novel therapeutic target for patients with CC.

## Background

Cervical cancer (CC) is a frequent malignancy hurting females all over the world [1]. According to previous reports, there are more than 529, 800 new CC patients every year, of which the mortality rate ratio is up to 50% [2]. At present, although it is achievable to reduce CC incidence and the mortality rates of CC patients though comprehensive methods contained effective screening, operation, chemotherapy as well as radiotherapy, the overall survival for patients with CC remains poor [3–5]. Among these methods, chemotherapy is less effective in treating patients with CC due to the obtained drug resistance of them after several sessions [6]. Also, because of the insufficient effective biomarker and limited diagnostic techniques, CC will continue to have high occurrence and recurrence rates in the coming decades. Recently, massive reports have pointed out that dysregulated lncRNAs play important roles in CC in terms of prognosis and tumor progression [7]. It provides a promising direction for treating CC. Hence, it is worthy doing more research on the molecular targets involving in CC development.

As a kind of non-coding RNAs, long non-coding RNAs (lncRNAs) exceeding 200 nucleotides present various modulatory roles like epigenetic, transcriptional, or post-transcriptional regulations [8]. Multiple studies have exhibited the close connection between maladjusted lncRNAs and the progression of cancers, including CC [9]. For instance, SNHG1, XIST and HOTAIR have been unveiled to regulate the biological behaviors of CC cells [10–12]. Hence, lncRNAs are naturally selected as crucial therapeutic targets for different cancers. As for aspartyl-tRNA synthetase antisense RNA 1 (DARS-AS1), it was previously found to actively involve into the progression of ovarian cancer [13], and also to facilitate malignant development in thyroid cancer [14]. Nonetheless, the function and regulatory way of DARS-AS1 in CC have not been illustrated yet.

In addition, growing evidence has showed that lncRNAs can function as competing endogenous RNAs (ceRNAs) to attenuate the suppression of microRNAs (miRNAs) on target genes, therefore regulating the expression of these genes and eventually affecting cancer development [15]. With regard to miRNAs mentioned in ceRNA network, a type of non-coding RNAs with a length about 20 nucleotides, it involves in post-transcriptional modulation to affect cancer progression by mediating biological behaviors of cells [16]. For instance, the importance of miR-628 has been validated in diverse cancer types [17]. So far, some studies have indicated that miRNAs participate in the progression of CC [18, 19]. Besides, miR-628-5p was explored and discovered to be a cancer-inhibitor in ovarian cancer [20] and prostate cancer [21]. However, its role and mechanism keep unknown and blurry in CC. Furthermore, jagged canonical Notch ligand 1 (JAG1), as a significant ligand of Notch signaling pathway, is firstly reported to be closely with breast cancer [22]. Importantly, it could interact with Notch receptor to activate Notch signaling pathway [23], while the significance of which in cancer development has already been disclosed [24]. Moreover, a study has revealed that lncRNAs can modulate Notch pathway activity by regulating the expression of its receptor and ligands [25]. Previously, the high association of JAG1 with the prognosis of CC patients has been unveiled [26]. However, it remains unknown whether DARS-AS1 can regulate JAG1 expression in CC.

In a nutshell, this study aimed at exploring the function and regulation mode of DARS-AS1 in CC. Hence, the functional impacts of DARS-AS1 silence on CC cell proliferation and apoptosis were determined. Furthermore, the molecular mechanism underlying DARS-AS1-regulated CC was dissected and probed.

## Methods

### Cell culture

Six kinds of CC cell lines (HeLa, C33A, MS751, SiHa, ME-180 and CaSki), as well as the normal cervical epithelial cell line (Ect1/E6E7), were all bought from the American Type Culture Collection (ATCC; Manassas, VA). Then, the culture medium for HeLa, C33A, MS751 and SiHa cells was Eagle’s Minimum Essential Medium (EMEM), and that for Ect1/E6E7 cells was Keratinocyte Serum Free Medium (K-SFM) (Gibco), while that for ME-180 and CaSki cells was McCoy’s 5a medium or RPMI-1640 medium, separately. All the media were supplemented with 10% FBS (Gibco). Cell culture was proceeded at 37 °C with 5% CO_2_.

### Real-time quantitative polymerase chain reaction (RT-qPCR)

The isolation of total RNA from transfected cells was processed with TRIzol reagent (Invitrogen, Carlsbad, CA). Following it, Reverse Transcription Kit (Thermo Scientific, Waltham, MA, USA) was adopted for cDNA synthesis. Next, SYBR-Green Real-Time PCR Kit (Qiagen, Zeeland, Netherlands) was exploited to carry out RT-qPCR. In the end, relative gene expression was computed by 2^−∆∆Ct^ method. The normalized control was GAPDH or U6. The primer sequences were provided in Table 1.

**Table 1:** The sequences of primers used in RT-qPCR

Primers	Sequences (5′–3′)
DARS-AS1-F	CATCGGGACACGGAACTGG
DARS-AS1-R	TGCAAAGAACTGCAGAAGACAC
miR-628-5p-F	GCCGAGATGCTGACATATTTAC
miR-628-5p-R	CTCAACTGGTGTCGTGGA
MGEA5-F	TGTGGCCAAAAGCATGATGG
MGEA5-R	GTACAAGAAAGTTGGGCACAGG
CACNB2-F	GAAGGCAATCATAGGCGAGC
CACNB2-R	GACCCTGCAGTACTCTGCTT
JAG1-F	CAAACACCAGCAGAAAGCCC
JAG1-R	TAAGTCAGCAACGGCCTCAG
ARRDC3-F	GAGACCACTGAGACGAGCG
ARRDC3-R	TTCACCTTTCCCAGCACCAT
VCAN-F	CTCGCAGAAACTGCATCACC
VCAN-R	CTGAGGTTGGACTGTGGCTT
GAPDH-F	CTCTGCTCCTCCTGTTCGAC
GAPDH-R	TTCCCGTTCTCAGCCTTGAC
U6-F	TCATCAGAAACAGTGGAGGT
U6-R	CATCCTTACACAGGAGCCAT

### Plasmid transfection

The short hairpin RNAs (shRNAs) specific to DARS-AS1 (sh-DARS-AS1#1/2) and the negative control (NC)-shRNAs (sh-NC), as well as miR-628-5p mimics/inhibitor and NC mimics/inhibitor, were devised and produced by GenePharma (Shanghai, China). Additionally, pcDNA3.1/DARS-AS1 and pcDNA3.1/JAG1 were gotten by inserting corresponding cDNA sequences into pcDNA3.1 vectors which were from Invitrogen. The transfection of above plasmids into HeLa and CaSki cells was realized with the aid of Lipofectamine 3000 (Invitrogen). After 48-h plasmid transfection, cells were collected and used for subsequent experiments. The target sequences of shRNAs were: sh-NC: 5′-CACCGTCTTTCAAGGATATGT-3′; sh-DARS-AS1#1: 5′-CACCGTCTGTTAGGGACACAT-3′; sh-DARS-AS1#2: 5′-CACCGTTTCTTTTAAGGAATG-3′.

### Cell counting kit-8 (CCK-8)

HeLa and CaSki cells were placed into 96-well plate for indicated time of incubation (24, 48 and 72 h). After that, cells in each plate were subjected to the culturing of 10 μl CCK-8 reagent (Dojindo Laboratories, Kumamoto, Japan) for 1 h. The microplate reader (EL340; Bio-Tek Instruments, Hopkinton, MA, USA) was exploited for testing the absorbance at 450 nm. Each experimental procedure was repeated for three times.

### Colony formation assay

After trypsin treatment, HeLa and CaSki cells were collected and then 800 cells were seed in each well of 6-well plates. Subsequently, cells were processed by culture medium which contained 10% FBS (Gibco) at RT. Two weeks later, the colonies were subjected to fixing and staining with methanol and 0.5% crystal violet (Sigma) respectively, followed by the calculation of visible colonies manually. Bio-repeats were run in triplicate.

### TdT-mediated dUTP Nick-End Labeling (TUNEL) assay

For measuring cell apoptotic capability, TUNEL assay was implemented according to the protocol of One-Step TUNEL Apoptosis Assay Kit (Beyotime. Shanghai, China). Moreover, the TUNEL-stained cells were observed through fluorescent microscope after the utilization of DAPI staining. Bio-repeats were run in triplicate.

### Flow cytometry analysis

After gathering the transfected HeLa and CaSki cells, the precooled PBS was adopted to rinse the cells. To measure cell apoptosis, Annexin V-FITC/PI Apoptosis kit obtained from BD Biosciences (San Jose, CA) was exploited. After culturing for 15 min in dark, cells were subjected to analysis with fortessa flow cytometry (BD Biosciences, CA. USA). Three experiments were conducted for each sample.

### Subcellular fractionation

According to the protocol of suppliers, the cytoplasm and nucleus of HeLa and CaSki cells were separated via Cytoplasmic and Nuclear RNA Purification Kit (Norgen, Ontario, Canada). Afterwards, RT-qPCR was implemented to estimate DARS-AS1 expression in two fractions. Besides, GAPDH acted as the cytoplasmic control and U6 as the nuclear control. The experiment was conducted in triplicate.

### FISH

After fixing with 4% formaldehyde, HeLa and CaSki cells were dehydrated and subsequently cultivated in hybridization buffer with DARS-AS1-FISH probe (Ribobio). Following DAPI staining, cells were then subjected to observation under a fluorescence microscope. The experiment was done in triplicate.

### RBP-JK luciferase reporter assay

Since RBP-JK [C protein binding factor 1/Suppressor of Hairless/Lag1 (CBF1/Su (H)/Lag 1)] is a transcription factor which directly regulates the downstream targets of Notch pathway [27], the activity of Notch signaling was assessed by RBP-JK luciferase reporter assays by use of the RBP-JK reporter kit (CCS-014L; SA Biosciences, Washington, USA). HeLa and CaSki cells in 96-well plates were subjected to the transfection of RBP-JK reporter with Lipofectamine 3000 (Invitrogen) based on the manufacturer’s instructions. 48 h later, the luciferase activity was monitored via Dual-Luciferase reporter system (Promega). The assay was carried out for three times.

### Western blot analysis

The extracted total proteins from HeLa and CaSki cells were subjected to separation by sodium dodecyl sulfate–polyacrylamide gel electrophoresis (SDS-PAGE), followed by transferring to the polyvinylidene difluoride (PVDF) membranes (Bio-Rad Laboratories). Following blockading with 5% nonfat milk, the membranes were subjected to cultivation with primary antibody for all night at 4 °C. After that, the membranes were rinsed in TBST, and secondary antibodies were supplemented for 1 h incubation at the room temperature. In the end, the bands were visualized through enhanced chemiluminescence (ECL; Millipore, USA). GAPDH was the internal control. Each sample was analyzed via western blots in triplicate for three times.

### RNA immunoprecipitation (RIP)

Depending on the protocol of suppliers, RIP assay was performed via Magna RNA-binding protein immunoprecipitation kit (Millipore, Bedford, MA). After supplementing RIP buffer, HeLa and CaSki cells were processed with Ago2 antibody (Millipore) or control IgG antibody (Millipore). Then, RT-qPCR was utilized for RNA analysis. RIP was conducted for three times.

### RNA pull down assay

First, the wild-type and mutated miR-628-5p sequence with or without sites complementary to DARS-AS1 or JAG1 sequence were composed and biotinylated into Bio-miR-628-5p-WT/Mut by Biotin RNA Labeling Mix (Roche, Mannheim, Germany), with a biotinylated nonsense sequence as the negative control (Bio-NC). Then, the biotinylated RNAs were separately incubated with cell lysates for one night. Afterwards, streptavidin-coupled agarose beads were supplemented for further 48 h of culture. In the end, RT-qPCR was employed for the detection of the purified RNA complex. Experiment was executed in triplicate.

### Luciferase reporter assay

Firstly, we utilized pmirGLO luciferase reporter vector (Promega, Madison, WI) to load DARS-AS1 or JAG1 3′UTR fragments with the wild/mutant-type target sequences for miR-628-5p, which were so-called pmirGLO/DARS-AS1-WT/Mut and pmirGLO/JAG1 3′UTR-WT/Mut. Then they were subjected to co-transfection for 48 h with miR-628-5p mimics or NC mimics. Finally, the mensuration of the luciferase activity in indicated HeLa and CaSki cells was achieved through dual luciferase reporter system (Promega). Luciferase reporter assay was implemented for three times.

### Statistical analyses

Each assay was implemented at least three times independently. The data were denoted as mean ± standard deviation (SD). Statistical analysis was conducted by GraphPad Prism 5.0 software (GraphPad Software, San Diego, CA). The differences of groups were estimated via Student’s t test or one-way analysis of variance (ANOVA). Besides, P < 0.05 represented statistical significance.

## Results

### DARS-AS1 functions as a tumor promoter in CC

**Fig. 1:**
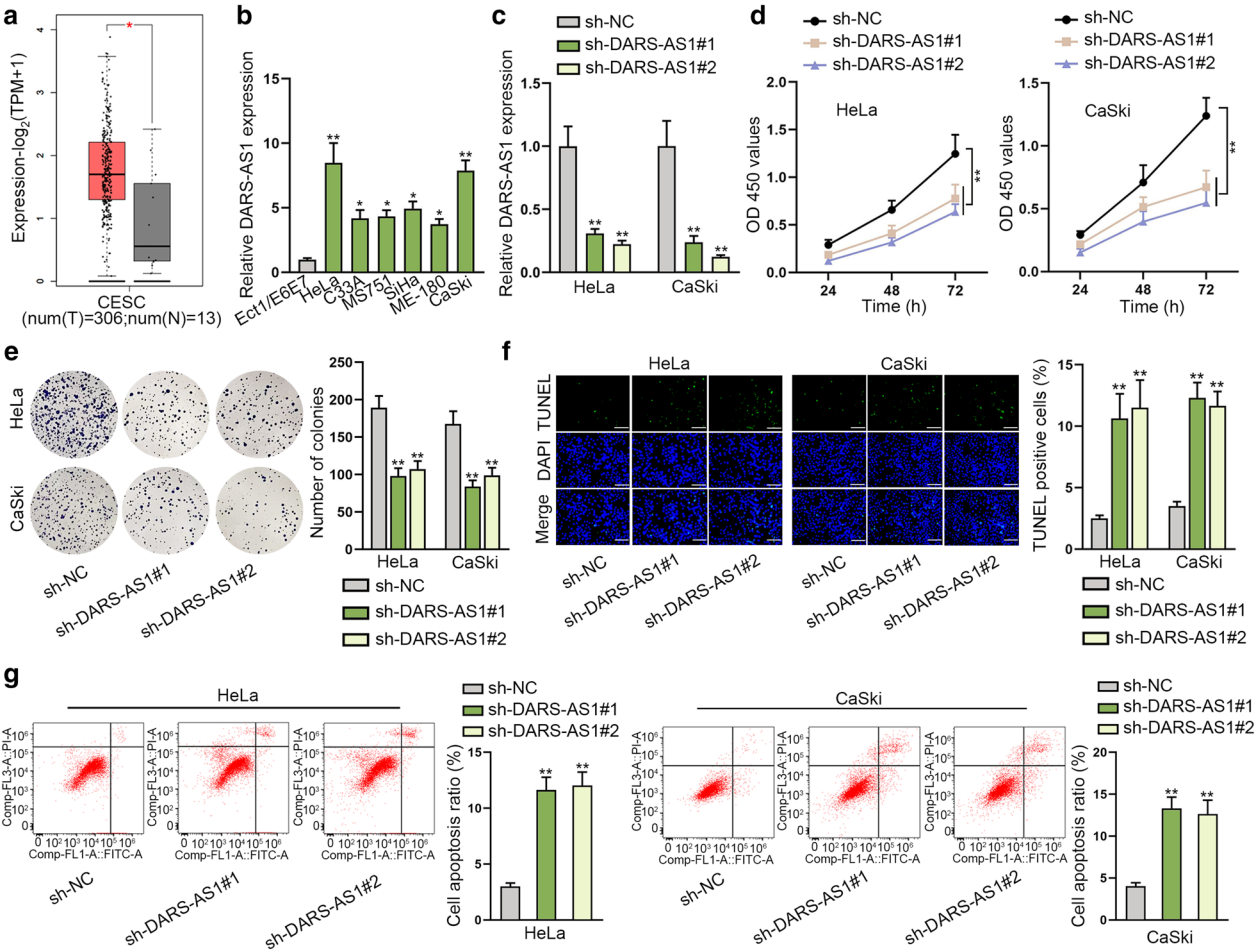
DARS-AS1 promotes cell proliferation and restrains cell apoptosis in CC. **a** GEPIA data indicated that DARS-AS1 level was enhanced in 306 CC tissues relative to 13 normal cervical tissues. **b** RT-qPCR proved that the expression of DARS-AS1 was elevated in CC cell lines (HeLa, C33A, MS751, SiHa ME-180 and CaSki) compared to human normal cervical epithelial cell line (Ect1/E6E7). **c** RT-qPCR evaluated the declined DARS-AS1 expression in HeLa and CaSki cells transfected with sh-DARS-AS1#1 or sh-DARS-AS1#2. **d** CCK-8 assay confirmed the suppressive effects of DARS-AS1 depletion on the viability of HeLa and CaSki cells. **e** Colony formation assay proved the proliferation capacities of HeLa and CaSki cells was impaired in the case of DARS-AS1 knockdown. **f** TUNEL assay detected that the apoptosis rate of HeLa and CaSki cells was heightened with DARS-AS1 inhibition. Scale bar = 100 μm. **g** Flow cytometry analysis assessed the promoted apoptosis ability of HeLa and CaSki cells in the case of DARS-AS1 knockdown. Data from three independent experiments were exhibited mean ± SD. *P < 0.05, **P < 0.01

In order to investigate the role of DARS-AS1 in CC, we first searched GEPIA database for whether it was differentially expression in tumor tissues. Interestingly, GEPIA data indicated an evident overexpression of DARS-AS1 in CESC (cervical squamous cell carcinoma and endocervical adenocarcinoma) samples compared to normal ones (Fig. 1a). In addition, we also detected the elevated expression of DARS-AS1 in CC cell lines (HeLa, C33A, MS751, SiHa, ME-180 and CaSki) relative to human normal cervical epithelial cell line (Ect1/E6E7) using RT-qPCR method (Fig. 1b). Therefore, we surmised that DARS-AS1 might play a key regulator in CC progression. To verify our conjecture, loss-of-function assays were carried out in HeLa and CaSki cells which possessed relatively higher expression of DARS-AS1 among all detected CC cell lines. Seen form Fig. 1c, RT-qPCR determined that in contrast to control group, the level of DARS-AS1 in HeLa and CaSki cells was seriously decreased after being transfected with sh-DARS-AS1#1 or sh-DARS-AS1#2. Subsequently, we observed that the viabilities of HeLa and CaSki cells were effectively inhibited in the context of DARS-AS1 knockdown (Fig. 1d). Likewise, the results from colony formation assay showed that DARS-AS1 silence declined the number of colonies compared with sh-NC group (Fig. 1e). Next, we further sought to explore the effect of DARS-AS1 on cell apoptosis by performing TUNEL assay and flow cytometry analysis. As presented in Fig. 1f, the results of TUNEL assay illustrated that positive stained cell ratio was overtly increased in these two CC cells under DARS-AS1 suppression. Similarly, flow cytometry analysis exhibited that cell apoptosis rate was obviously enhanced when DARS-AS1 was down-regulated (Fig. 1g). Based on above data, we could learn that DARS-AS1 plays as a tumor-promoter in CC.

### DARS-AS1 interacts with miR-628-5p and also functions depending on miR-628-5p in CC

**Fig. 2:**
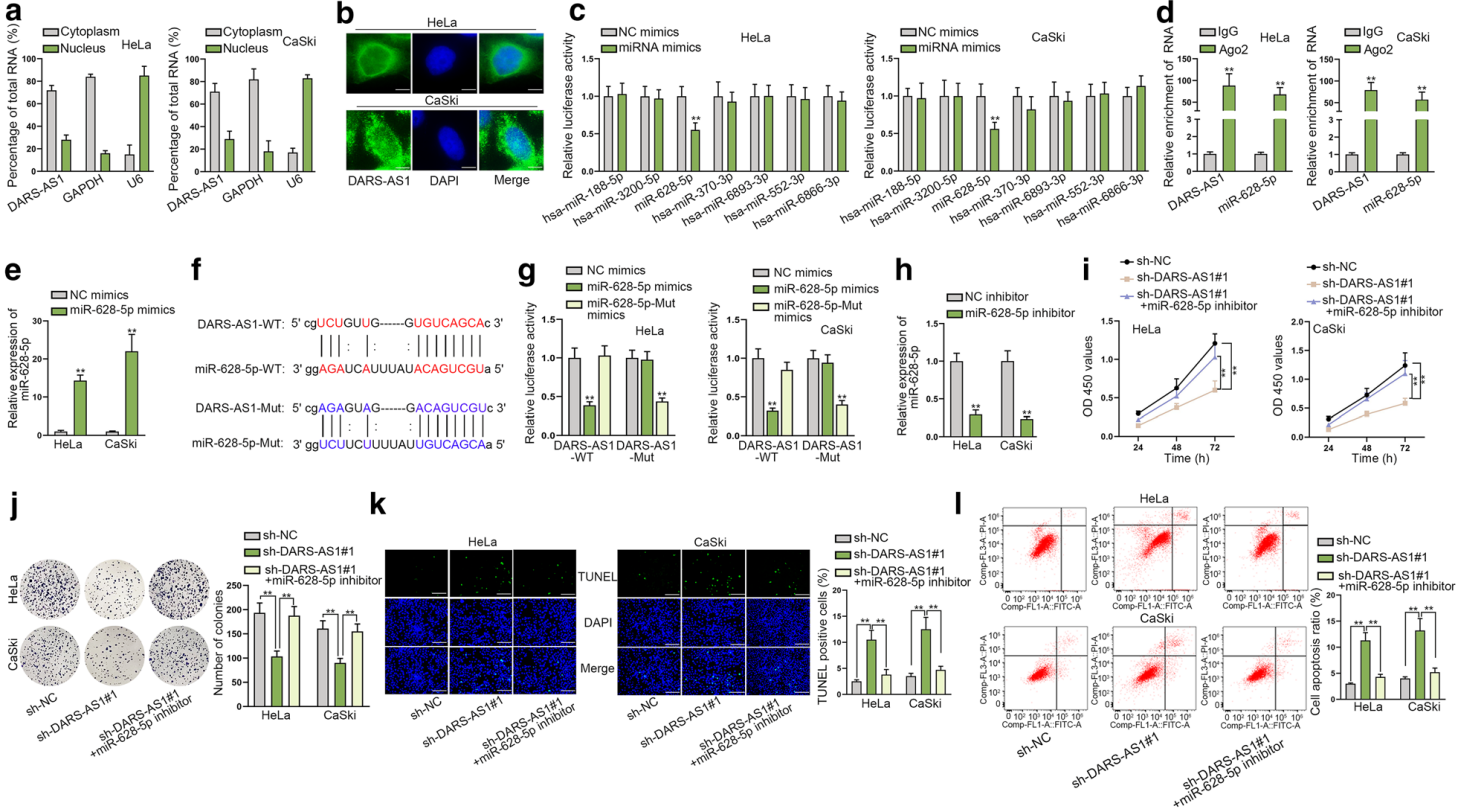
DARS-AS1 acts as a sponge of miR-628-5p in CC cells. **a** Subcellular fractionation assay showed the main cytoplasmic location of DARS-AS1 in CC cells. **b** FISH assay was determined that DARS-AS1 was primarily located in the cytoplasm of CC cells. Scale bar = 10 μm. **c** Luciferase reporter assay found the specifically decreased luciferase activity of DARS-AS1 under the upregulation of miR-628-5p rather than other miRNAs. **d** Ago2-RIP assay followed by RT-qPCR confirmed the high enrichment of DARS-AS1 and miR-628-5p in Ago2-assembled RISC. **e** The successful overexpression of miR-628-5p was tested via RT-qPCR. **f** The binding sites between wild-type DARS-AS1 and miR-628-5p (DARS-AS1-WT and miR-628-5p-WT) were predicated by ENCORI, and the sequences of mutant DARS-AS1 and miR-628-5p (DARS-AS1-Mut and miR-628-5p-Mut) were designed. **g** Luciferase reporter assay validated the specific combination between DARS-AS1 and miR-628-5p at the predicted sites. **h** RT-qPCR verified the evident interference of miR-628-5p in two CC cells by miR-628-5p inhibitor. **i** CCK-8 assay found that the declined viabilities of cells under DARS-AS1 suppression were recovered after co-transfected with sh-DARS-AS1#1 and miR-628-5p inhibitor. **j** Colony formation assay assessed that cell proliferation capacities hindered by sh-DARS-AS1#1 was then reversed under the co-inhibition of miR-628-5p. **k**, **l** TUNEL assay (scale bar = 100 μm) and flow cytometry analysis unveiled that DARS-AS1 silence-promoted cell apoptosis was cut down in response to further inhibition of miR-628-5p. The data from three independent experiments were presented as mean ± SD. **P < 0.01

To further explore the possible molecular mechanism of DARS-AS1 in CC, subcellular fractionation assay and FISH assay were carried out. As a result, DARS-AS1 was uncovered to be abundant in the cytoplasm of HeLa and CaSki cells (Fig. 2a, b), implying the possibility for DARS-AS1 to work at the post-transcriptional level. To identify downstream miRNAs that might own interplays with DARS-AS1, ENCORI (http://starbase.sysu.edu.cn/) database was employed and then 7 candidates (miR-188-5p, miR-3200-5p, miR-628-5p, miR-370-3p, miR-6893-3p, miR-553-3p and miR-6866-3p) were screened out. Further, we discovered that the luciferase activity of DARS-AS1 was visibly inhibited by elevated miR-628-5p, while the changes of it were not significant after upregulating other miRNAs (Fig. 2c). Therefore, miR-628-5p was selected for later experiments. To further identify the relationship between DARS-AS1 and miR-628-5p in CC, we performed Ago2-RIP assay. The outcome demonstrated that DARS-AS1 and miR-628-5p were both remarkably harvested in Ago2 group instead of IgG group (Fig. 2d), indicating they were co-existed in RNA-induced silencing complexes (RISCs). Then, we elevated miR-628-5p expression in HeLa and CaSki cells by transfecting with miR-628-5p mimics (Fig. 2e). As presented in Fig. 2f, the binding sites between DARS-AS1 and miR-628-5p predicted by ENCORI, as well as the mutant DARS-AS1 and miR-628-5p with the putative binding sites mutated, were shown. As expected, the outcomes of luciferase reporter assay suggested that the luciferase activity of pmirGLO/DARS-AS1-WT was suppressed only by miR-628-5p mimics, while that of pmirGLO/DARS-AS1-Mut could also be decreased only by enhanced level of mutant miR-628-5p (Fig. 2g), highlighting the specific interaction between DARS-AS1 and miR-628-5p at the predicted binding sites. These data illustrated that DARS-AS1 could combine with miR-628-5p in CC cells.

Wondering whether the DARS-AS1/miR-628-5p axis affected the biological behaviors of CC cells, we designed corresponding rescue experiments in HeLa and CaSki cells. Before this, we verified the efficient interference of miR-628-5p in these two cells by miR-628-5p inhibitor (Fig. 2h). Subsequently, the results of CCK-8 assay uncovered that miR-628-5p down-regulation in CC cells restored DARS-AS1 depletion-mediated loss of cell viability (Fig. 2i). Also, we observed that miR-628-5p silence in CC cells recovered the hindered proliferation under DARS-AS1 knockdown (Fig. 2j). Meanwhile, we also unveiled that the apoptosis increase caused by DARS-AS1 depletion was absolutely abolished by miR-628-5p inhibitor (Fig. 2k, l). Taken together, DARS-AS1 sponges miR-528-5p to facilitate CC development.

### DARS-AS1 modulates JAG1 to activate Notch pathway by binding with miR-628-5p

**Fig. 3:**
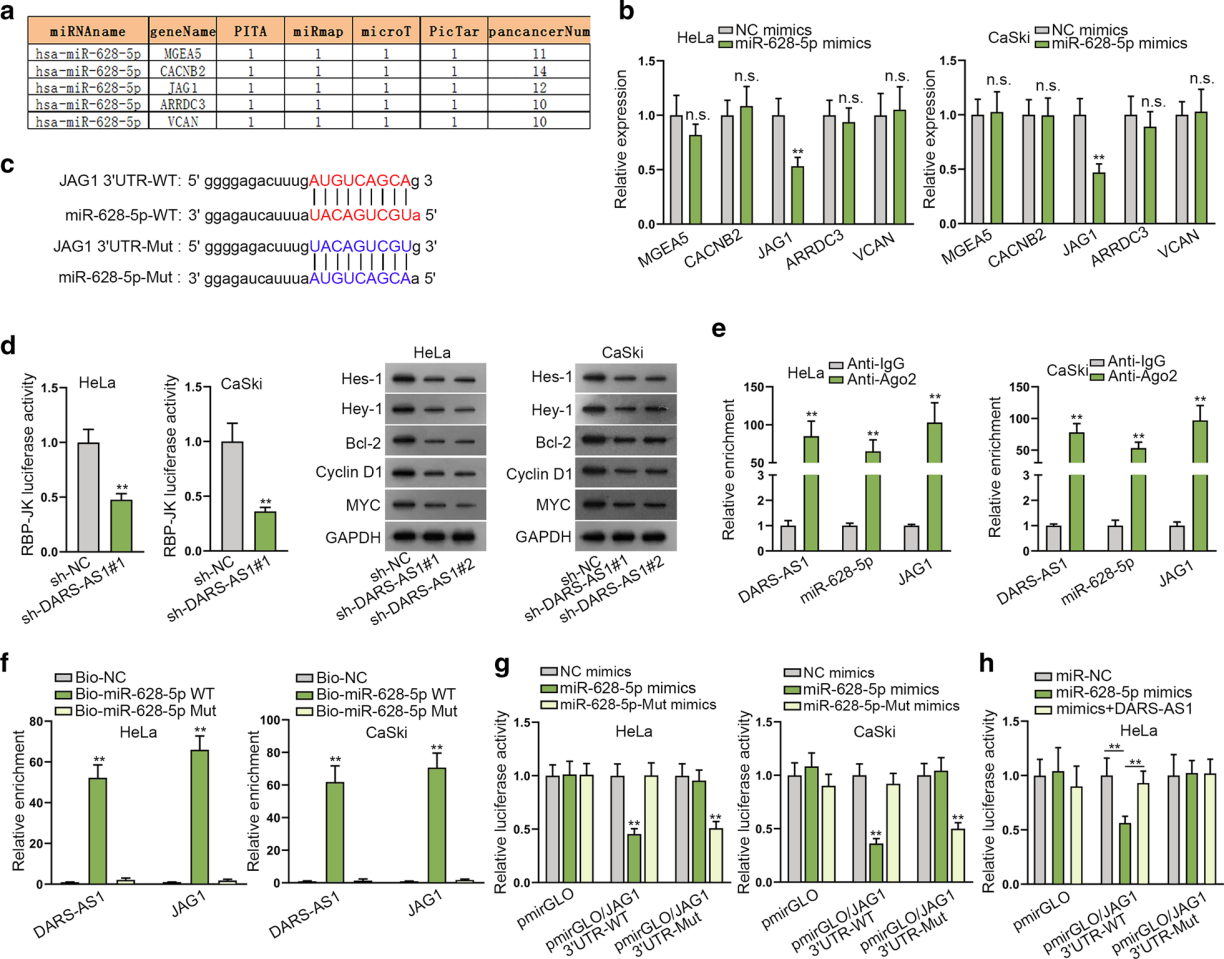
DARS-AS1 modulates JAG1 to activate Notch pathway by binding with miR-628-5p. **a** ENCORI predicted 5 potential target genes of miR-628-5p. **b** RT-qPCR unveiled that only the expression levels of JAG1 was markedly reduced in CC cells transfected with miR-628-5p mimics. **c** The binding sites between miR-628-5p-WT and JAG1-WT were exhibited by ENCORI, and the sequences of miR-628-5p-Mut and JAG1-Mut were designed. **d** RBP-JK luciferase reporter assay and western blot analysis confirmed the inactivation of DARS-AS1 depletion on Notch pathway. **e** RIP assay followed by RT-qPCR confirmed the strong enrichment of DARS-AS1, miR-628-5p and JAG1 in Ago2-involved RISC in CC cells. **f** RNA pull down assay disclosed the high enrichment of DARS-AS1 and JAG1 in Bio-miR-628-5p-WT groups in CC cells. **g** The combination between JAG1 and miR-628-5p at predicted binding sites was verified via luciferase reporter assay. **h** Luciferase reporter assay determined that DARS-AS1 competed with JAG1 to interact with miR-628-5p in CC cells. All data were displayed as the mean ± SD from three independent experiments. **P < 0.01, n.s.: no significance

To expose the downstream of DARS-AS1/miR-628-5p signaling in CC, we utilized ENCORI to predict the underlying targets of miR-628-5p. By screening miRmap, PITA, microT and PicTar datasets, five probable miR-628-5p targets including MGEA5, CACNB2, JAG1, ARRDC3 and VCAN were ultimately sifted out (Fig. 3a). Thereafter, RT-qPCR analyzed that miR-628-5p overexpression just declined the expression level of JAG1, while didn’t affect that of other candidates (Fig. 3b). Hence, JAG1 was chosen as the possible target of miR-628-5p in CC. In addition, ENCORI predicted the binding sites between miR-628-5p and JAG1, and the mutant miR-628-5p and JAG1 sequences containing mutated binding sites were also exhibited (Fig. 3c). Given that JAG1 was a key ligand in Notch signaling pathway, we hypothesized DARS-AS1 affected Notch signaling pathway by regulating JAG1. As anticipated, the RBP-JK luciferase activity was effectively suppressed, and the protein levels of several JAG1 downstream genes (including Hes-1, Hey-1, Bcl-2, Cyclin D1 and MYC) were declined, in both HeLa and by DARS-AS1 silence (Fig. 3d), implying that DARS-AS1 knockdown made Notch signaling pathway inactivated. Furthermore, RIP and RNA pull down as well as luciferase reporter assay were implemented in succession to determine the association among DARS-AS1, miR-628-5p and JAG1. The outcome of RIP assay illustrated that DARS-AS1, miR-628-5p and JAG1 were concentrated in anti-Ago2 complex rather than anti-IgG complex (Fig. 3e), indicating that these three RNAs were in RISC. Meanwhile, RNA pull down assay results showed that only Bio-miR-628-5p-WT, not Bio-miR-628-5p-Mut, could capture DARS-AS1 and JAG1 abundantly relative to Bio-NC group (Fig. 3f). According to the results of luciferase reporter assay, we observed that elevating the expression of wild-type miR-628-5p declined the luciferase activity of pmirGLO/JAG1 3′UTR-WT but didn’t impact that of pmirGLO/JAG1 3′UTR-Mut, whereas ectopic expression of miR-628-5p-Mut led to reduced luciferase activity of pmirGLO/JAG1 3′UTR-Mut but not pmirGLO/JAG1 3′UTR-WT (Fig. 3g) indicating the binding specificity between JAG1 and miR-628-5p at the putative sites. In the end, we further certified that the luciferase activity of pmirGLO/JAG1 3′UTR-WT lowered by miR-628-5p elevation, was then recovered after DARS-AS1 up-regulation (Fig. 3h). Taken together, DARS-AS1 boosts JAG1 to activate Notch pathway by competitively binding with miR-628-5p in CC cells.

### DARS-AS1 promotes cell proliferation and restrains cell apoptosis by up-regulating JAG1

**Fig. 4:**
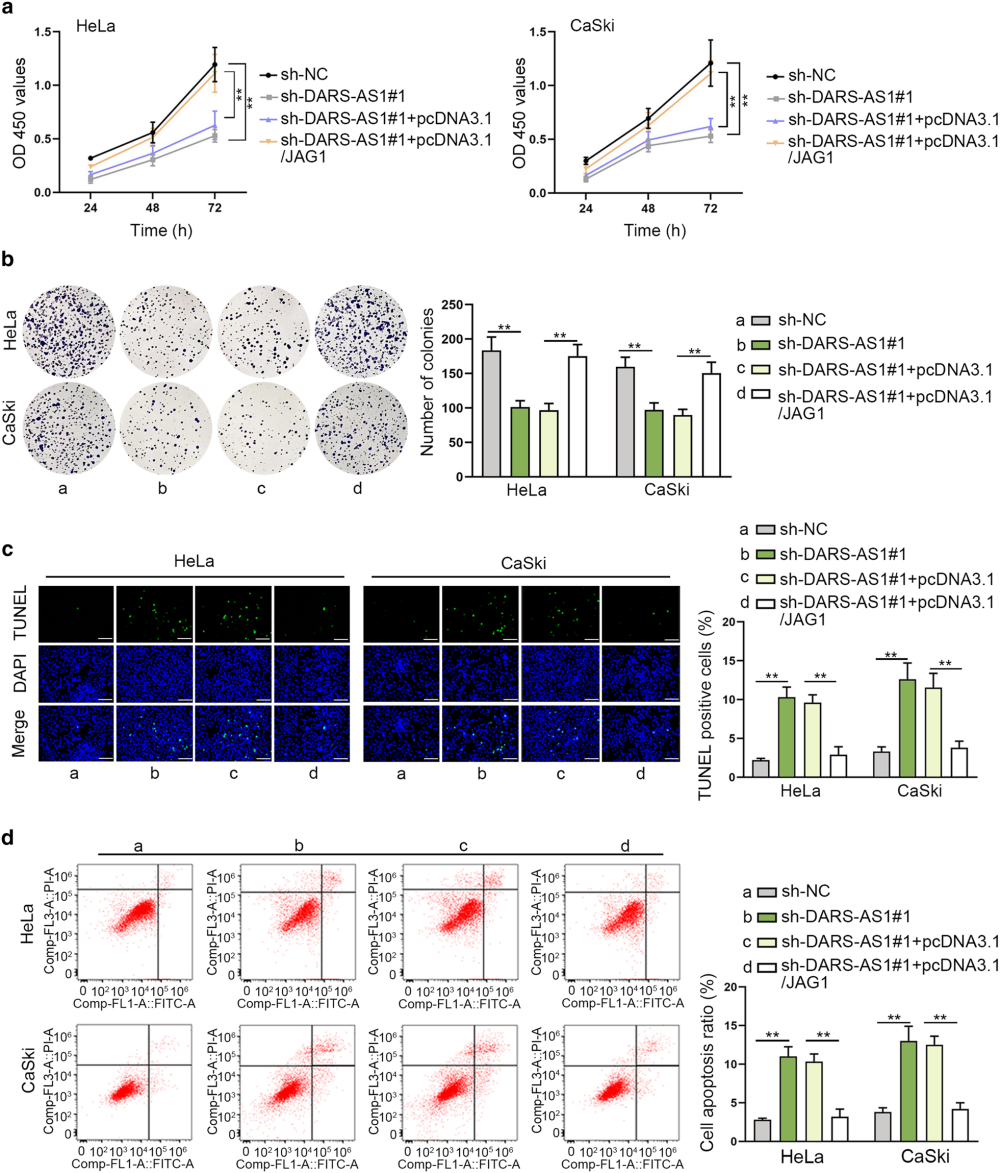
DARS-AS1 depends on JAG1 to regulate the proliferation and apoptosis of CC cells. **a**, **b** CCK-8 and colony formation assays validated that the restrained proliferation of CC cells induced by DARS-AS1 deficiency was recovered under JAG1 overexpression. **c**, **d** TUNEL assay (scale bar = 100 μm) and flow apoptosis analysis certified that cell apoptosis stimulated in the case of DARS-AS1 silence was normalized under JAG1 upregulation. The data of three independent experiments were presented as mean ± SD. **P < 0.01

**Fig. 5:**
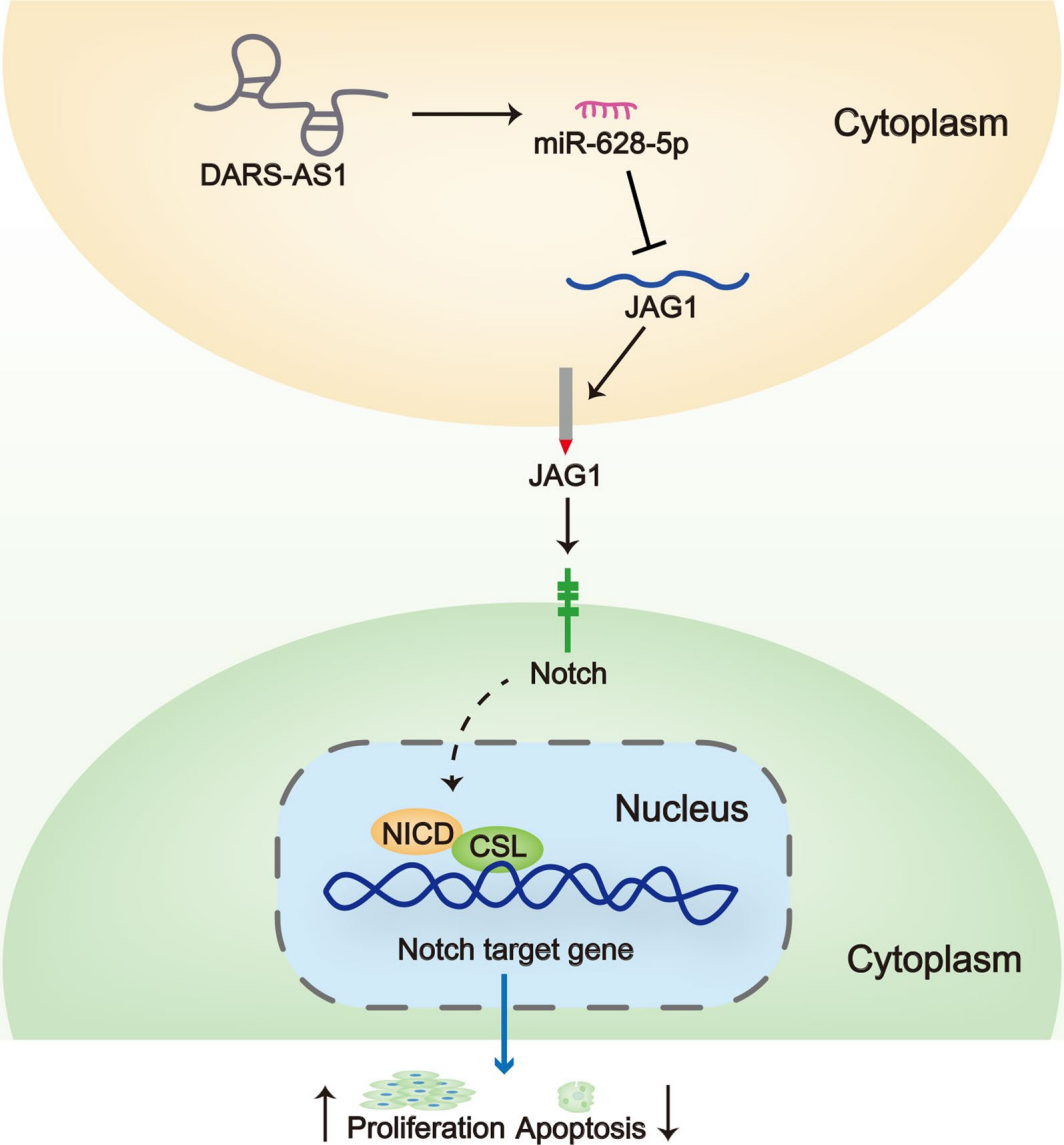
The diagram illustrated that DARS-AS1/miR-628-5p/JAG1 axis promotes the proliferation of cervical cancer cells by activating Notch signaling pathway

In the final steps, to further determine whether JAG1 was responsible for the function of DARS-AS1 in CC cells, the rescue assays were designed and then implemented in HeLa and CaSki cells transfected with sh-NC, sh-DARS-AS1#1, sh-DARS-AS1#1 + pcDNA3.1 orsh-DARS-AS1#1 + pcDNA3.1/JAG1. Next, we conducted CCK-8 and colony formation assay to assess the alteration of cell proliferation. As anticipated, the inhibition on cell viability caused by silenced DARS-AS1 was fully rescued by JAG1 up-regulation (Fig. 4a). Similarly, the results of colony formation assay also uncovered that the number of colonies decreased by DARS-AS1 silence was then restored by JAG1 increase (Fig. 4b). These data indicated that DARS-AS1 promoted cell proliferation by up-regulating JAG1. Finally, to confirm the effect of DARS-AS1/JAG1 axis on cell apoptosis, the TUNEL assay and flow cytometry were performed. As displayed in Fig. 4c, the ratio of TUNEL positive stained cells was elevated under DARS-AS1 depletion, while it was subsequently declined with the increase of JAG1. Likewise, the results of flow cytometry analysis indicated that JAG1 overexpression abolished the enhancing effect of DARS-AS1 silence on cell apoptosis (Fig. 4d). In summary, DARS-AS1 sponges miR-628-5p to enhance JAG1 expression in cytoplasm, so as to activate Notch pathway to facilitate CC cell proliferation and hinder cell apoptosis (Fig. 5).

## Discussion

Growing reports have unveiled that lncRNAs are closely related to cancer progression [28]. In present study, high expression of DARS-AS1 exhibited a tumor-promoting function in CC. Similarly, many earlier studies have indicated that some lncRNAs acted as oncogenes or cancer suppressors in CC [29]. For instance, Shan et al. discovered that lncRNA CASC15 expression is abnormally high in CC cells and it predicts poor prognosis [30]. In this study, we found that DARS-AS1 was elevated in CC tissues and also proved its upregulation in CC cells. Moreover, we unveiled that loss of DARS-AS1 inhibited CC cell proliferation and inducted cell apoptosis. In other word, DARS-AS1 worked as a cancer promoter in CC, which was consistent with its role in several other cancer types, such as non-small cell lung cancer [31], myeloma [32], and clear cell renal cell carcinoma [33]. Collectively, DARS-AS1 could be developed as a novel target for the treatment of CC.

Numerous studies have validated the mutual interaction between lncRNAs and miRNAs, which may finally affect cell proliferation and apoptosis, or even metastasis in diverse tumors [34]. In this study, miR-628-5p was finally identified as the downstream factor of DARS-AS1 in CC. Rescue experiments further identified that miR-628-5p inhibition abolished the effect of DARS-AS1 depletion on CC cell proliferation and apoptosis, suggesting miR-628-5p as a cancer repressor in CC. Likewise, former literatures uncovered that upregulation of miR-628-5p suppresses the proliferation of glioma cells [35], while loss of miR-628-5p expedites the development of gastric cancer [36], both of which reflected miR-628-5p as a cancer repressor. Furthermore, miRNAs have been confirmed to degrade the expression of genes whose 3′UTR was recognized by such miRNAs [37]. In current study, JAG1, a Notch ligand that activates Notch pathway and therefore affect disease development [38], was confirmed to be the target of miR-628-5p. Unsurprisingly, this study found that DARS-AS1 activated the Notch signaling pathway in CC cells by positively modulating JAG1. Furthermore, the results of rescue assays validated that the inhibitory effect of DARS-AS1 silence on CC cell growth was counteracted by JAG1 overexpression. Taken together, DARS-AS1 accelerated CC tumorigenesis by targeting miR-628-5p/JAG1/Notch pathway. The similar mechanisms upstream of JAG1 have been exhibited by studies focusing on other cancers. For instance, JAG1 is modulated by MALAT1/miR-125 signaling to promote tongue cancer development [39]. Besides, the discovery of DANCR/miR-34a-5p/JAG1 axis provides a novel perspective on the treatment of prostate cancer [40].

In summary, this study indicated that DARS-AS1, highly-expressed in CC, contributes to the CC tumorigenesis through activating Notch signaling via miR-628-5p/JAG1 pathway. Based on this, we may suggest that targeting DARS-AS1 is possibly an effective therapeutic avenue for patients with CC. In the end, we need to emphasize that although the discovery of the ceRNA network (DARS-AS1/miR-628-5p/JAG1 signaling) provides a promising direction, the significance of DARS-AS1 in CC needs to be further explored in further research, especially large-scale clinical study.

## Conclusion

This study indicated that DARS-AS1, highly-expressed in CC, contributes to the CC tumorigenesis through activating Notch signaling via miR-628-5p/JAG1 pathway. Based on this, we may suggest that targeting DARS-AS1 is possibly an effective therapeutic avenue for patients with CC. In the end, we need to emphasize that although the discovery of the ceRNA network (DARS-AS1/miR-628-5p/JAG1 signaling) provides a promising direction, the significance of DARS-AS1 in CC needs to be further explored in further research, especially large-scale clinical study.

## Data Availability

Research data and materials have been provided in current manuscript.

## Abbreviations

lncRNAs: Long non-coding RNAs
ceRNAs: Competing endogenous RNAs
miRNA: MicroRNA
mRNA: Messenger RNA
CC: Cervical cancer
DARS-AS1: Aspartyl-tRNA synthetase antisense RNA 1
JAG1: Jagged canonical Notch ligand 1
ATCC: American Type Culture Collection
EMEM: Eagle’s Minimum Essential Medium
K-SFM: Keratinocyte Serum Free Medium
RPMI: Roswell Park Memorial Institute
FBS: Fatal Bovine Serum
DAPI: 4′,6-diamidino-2-phenylindole
RT-qPCR: Real-time quantitative polymerase chain reaction
CCK-8: Cell counting kit-8
TUNEL: TdT-mediated dUTP Nick-End Labeling
FISH: Fluorescence in situ hybridization
RIP: RNA immunoprecipitation
SDS-PAGE: Sodium dodecyl sulfate–polyacrylamide gel electrophoresis
PVDF: Polyvinylidene difluoride membranes
WT: Wild-type
Mut: Mutant
SD: Standard deviation
ANOVA: Analysis of variance
